# Egg consumption improves vascular and gut microbiota function without increasing inflammatory, metabolic, and oxidative stress markers

**DOI:** 10.1002/fsn3.2671

**Published:** 2021-11-30

**Authors:** Xiang Liu, Yijia Shao, Jiapan Sun, Jiazichao Tu, Zhichao Wang, Jun Tao, Jimei Chen

**Affiliations:** ^1^ Department of Cardiac Surgery Guangdong Cardiovascular Institute Guangdong Provincial People’s Hospital Guangdong Academy of Medical Sciences Guangzhou China; ^2^ Guangdong Provincial Key Laboratory of South China Structural Heart Disease Guangzhou China; ^3^ School of Medicine South China University of Technology Guangzhou China; ^4^ Department of Hypertension and Vascular Diseases The First Affiliated Hospital Sun Yat‐sen University Guangzhou China; ^5^ NHC Key Laboratory of Assisted Circulation (Sun Yat‐sen University) Guangzhou China; ^6^ Department of Geriatrics Peking University Shenzhen Hospital Shenzhen Peking University‐The Hong Kong University of Science and Technology Medical Center Shenzhen China

**Keywords:** egg consumption, gut microbiota, trimethylamine‐N‐oxide, vascular function

## Abstract

Egg consumption is one of the many inconsistencies in evidence linking dietary cholesterol to cardiovascular disease (CVD). In addition, the gut microbiota and its metabolite, trimethylamine‐N‐oxide (TMAO), have been shown to play a crucial role in the development of CVD. The fact that egg is rich in choline suggests that excessive egg consumption may increase TMAO production by altering the gut microbiota. However, the effects of egg consumption on vascular function and gut microbiota remain unclear. Here, the diet of nine young male subjects was supplemented with two boiled eggs daily for 2 weeks. Changes in vascular function, inflammation, metabolism, oxidative stress, and gut microbiota were examined. We found that egg consumption increased flow‐mediated dilation and decreased brachial‐ankle pulse wave velocity. Furthermore, egg consumption positively modulated the gut microbiota function but had no effects on the levels of C‐reactive protein, glucose, lipid profile, malondialdehyde, superoxide dismutase, or TMAO. The current study provides evidence that egg consumption improves vascular function, which may be related to the alterations seen in the gut microbiota. Therefore, moderate egg consumption may help to improve vascular and intestinal function in individuals at low risk of developing CVD and other metabolic disorders.

## Graphical abstract

Egg consumption improves vascular and gut microbiota function but has no effects on inflammatory, metabolic, and oxidative status.

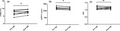

## 1 INTRODUCTION

Cardiovascular disease (CVD) remains a major long‐term public health challenge globally (Roth et al., 2020). Hyperlipidemia has been demonstrated to be a preventable risk factor for CVD (Hong et al., 2017). Previous guidelines stated that cholesterol intake should be limited to achieve a healthy lipid profile (Carson et al., 2020). However, current dietary guidelines have removed the upper limit of dietary cholesterol intake due to inconsistencies in evidence linking increased dietary cholesterol intake to elevated CVD risk (Soliman, 2018). Active debate surrounding these guidelines remains in the literature due to the dogma that the rich cholesterol content of eggs leads to increased CVD risk.

In recent years, the gut microbiota has received widespread interest (Fan & Pedersen, 2021; Marchesi et al., 2016). Changes in the composition and function of the gut microbiota are associated with CVD, including atherosclerosis, hypertension, and heart failure (Tang et al., 2017). Dietary choline is metabolized into trimethylamine (TMA) via a microbiota‐dependent mechanism and then catalyzed into trimethylamine‐N‐oxide (TMAO) in the liver (Wang et al., 2011). As one of the most important metabolites of the intestinal flora, TMAO has been identified as a novel biomarker for CVD (Tang et al., 2013; Wang et al., 2011) and as a crucial player in the development and progression of atherosclerosis, endothelial dysfunction, cardiac fibrosis, and metabolic dysfunction (Chen et al., 2019; Ke et al., 2018; Wang et al., 2011; Wu, Chen et al., 2020; Yang et al., 2019). It may be reasonable to speculate that since eggs are rich in choline, excessive consumption of eggs may alter the gut microbiota and increase TMAO production. However, the effects of egg consumption on vascular function and gut microbiota in healthy adults remain unclear.

In this study, the diets of nine young male subjects were supplemented with two boiled eggs daily for 2 weeks. Changes in vascular function, inflammation, metabolism, oxidative stress, and gut microbiota were examined.

## 2 MATERIALS AND METHODS

### 2.1 Experimental design, study population, and egg intervention

This was a prospective nonrandomized intervention trial designed to evaluate the effects of daily intake of two eggs on vascular function, inflammatory, metabolic and oxidative status, and gut microbiota. Twelve healthy male volunteers were recruited from the university, who usually ate in the cafeteria where regular and simple diet formula was supplied. These volunteers were all male college students and above 18 years old. Comprehensive medical history was obtained, and physical, laboratory, and imaging examinations were performed. Subjects with CVD, hyperlipidemia, diabetes, infectious diseases, severe traumas, or who underwent operations during the early part of the previous month were excluded. Each consumed two eggs per day for 2 weeks without changing their usual diet. Whole eggs were fully cooked in boiling water and supplied to the volunteers by the researcher every morning. Two eggs weighed about 90–100 g and contained around 460–500 mg of cholesterol. This study was approved by the Hospital Ethics Committee (No. 202059601), and informed consent was obtained from all subjects. This trial was registered at the Chinese Clinical Trial Registry (ChiCTR2100046956).

### 2.2 Blood collection, processing, and detection

Whole blood was collected from participants at baseline and at the end of the trial. After a 12 hr overnight fast, blood was drawn into EDTA and SST blood collection tubes and centrifuged at   1811 × *g* for 10 min at 4°C, and the plasma was collected. Levels of biochemical, lipid profile, and C‐reactive protein (CRP) were tested in the clinical laboratory of the First Affiliated Hospital, Sun Yat‐sen University. The levels of malondialdehyde (MDA) and superoxide dismutase (SOD) in the blood were measured using Lipid Peroxidation MDA Assay Kit (Beyotime) and Total Superoxide Dismutase Assay Kit with WST‐8 (Beyotime) respectively, which were all performed according to the standard protocols recommended by the manufacturer.

### 2.3 Measurements of flow‐mediated dilation, brachial‐ankle pulse wave velocity, and ankle‐brachial index

All the procedures were performed by the same skilled technician. Flow‐mediated dilation (FMD) was performed on a high‐resolution ultrasonography equipment (UNEXEF18G; UNEX Co) as described in our previous study (Zhang et al., 2021). Briefly, after overnight fasting, subjects were placed in a supine position in a temperature‐controlled room (22°C). The diameter of the brachial artery was automatically imaged using a high‐resolution linear artery transducer. A blood pressure cuff was placed around the forearm, and the brachial artery was visualized 5–10 cm above the right cubital crease. Pulsed Doppler flow was measured at baseline and during peak hyperemic flow. The traverse images of the artery at baseline were acquired for 30 s, and then the cuff was inflated up to a pressure of 50 mmHg higher than systolic pressure, and maintained for 5 min. Cross‐sectional images of the artery were recorded continuously until 1 min after the cuff was deflated. FMD (%) was calculated using the following formula: (peak diameter‐baseline diameter)/baseline diameter × 100%. Subject having an FMD equal to or less than 7% indicates endothelial dysfunction.

Measurements of brachial‐ankle pulse wave velocity (baPWV) and ankle‐brachial index (ABI) were performed using an automatic measuring instrument (Colin Co. Ltd.) as described in our previous study (Yang et al., 2021). The analyzer can record PWV, blood pressure (BP), electrocardiogram (ECG), heart sounds, and ABI simultaneously. Briefly, after overnight fasting, subjects were placed in a supine position in a temperature‐controlled room (22°C). ECG electrodes were placed on both wrists, cuffs were placed on both the brachia and ankles, and a microphone was placed on the left edge of the sternum. The baPWV on each side was automatically calculated, and the average value of both the sides was used for subsequent analysis.

### 2.4 Concentrations of trimethylamine‐N‐oxide

The concentrations of TMAO in the fasting plasma and urine were detected on a liquid chromatography‐tandem mass spectrometry (LC‐MS/MS) system consisting of an Agilent 1260 high‐performance liquid chromatography (HPLC) and 6420 triple quadrupole mass spectrometer with an electrospray ionization source (ESI) (Agilent). One hundred microliters of the specimen was mixed with internal standard (IS) working solution (d9‐TMAO), and 300 μL of acetonitrile was added to the sample and then vortexed, and the mixture was centrifuged at  15,294 × *g* for 5 min at 4°C. Finally, the supernatant was transferred into an autosampler vial, and 2 μL was injected into the HPLC‐MS/MS for analysis. Chromatographic separation was performed on the Waters Atlantis HILIC Silica column (3.0 × 100 mm, 3.0 μm), which was equilibrated with 30% solution A (10 mmol/L ammonium formate in water, pH 3.0) and 70% solution B (acetonitrile) under isocratic elution with the flow rate of 0.3 ml/min. The ESI was operated in positive mode, and the mass spectrometer was run in multiple‐reaction monitoring mode. The settings for the mass transition of TMAO and IS were *m*/*z* 76.1 → 58.3 and *m*/*z* 85.1 → 66.3.

### 2.5 Fecal sample collection, 16S rRNA gene sequencing, and analysis

Fecal samples were collected from every participant at baseline and at the end of the trial and then frozen at −20°C for subsequent analysis. DNA was extracted using the GHFDE100 DNA isolation kit according to the manufacturer's protocol. PCR amplification of the bacterial 16S rDNA genes V4 region was performed using a Phusion High‐Fidelity PCR Master Mix (New England Biolabs) with the forward primer 515F (5'‐GTGCCAGCMGCCGCGGTAA‐3') and the reverse primer 806R (5'‐GGACTACHVGGGTWTCTAAT‐3'). Next, PCR amplicons were purified with Agencourt AMPure XP Beads (Beckman Coulter) and quantified using the PicoGreen dsDNA Assay Kit (Invitrogen). Finally, the sequencing was performed using the Illumina NovaSeq 6000 platform.

The sequencing data were processed using the Quantitative Insights Into Microbial Ecology (QIIME2, v2020.6) pipeline (Bolyen et al., 2019). Venn diagram was generated to visualize the shared and unique operational taxonomic units (OTUs) between groups using VennDiagram. Alpha diversity (Shannon, Simpson, Chao1) was calculated in QIIME2. Beta diversity was visualized via nonmetric multidimensional scaling (NMDS) (Ramette, 2007) and further analyzed by analysis of similarities. Microbial functions were predicted by the Phylogenetic Investigation of Communities by Reconstruction of Unobserved States (PICRUSt2) and also analyzed using human gut metabolic modules (GMMs) (Vieira‐Silva et al., 2016).

### 2.6 Statistical analyses

Statistical analysis was conducted using the SPSS 20.0 software (SPSS Inc.), and the graphs were plotted by GraphPad Prism (Version 7.0). Continuous variables with normal distribution were presented as mean ± standard error of the mean, and the comparisons between two groups were performed with the paired *t* test. Continuous variables with non‐normal distribution were presented as median and interquartile range, and the comparisons between two groups were performed with the Wilcoxon rank‐sum test. Categorical variables were described as whole numbers and percentages. *p* < .05 was considered statistically significant.

## 3 RESULTS

### 3.1 Subject characteristics and laboratory indices

**FIGURE 1:**
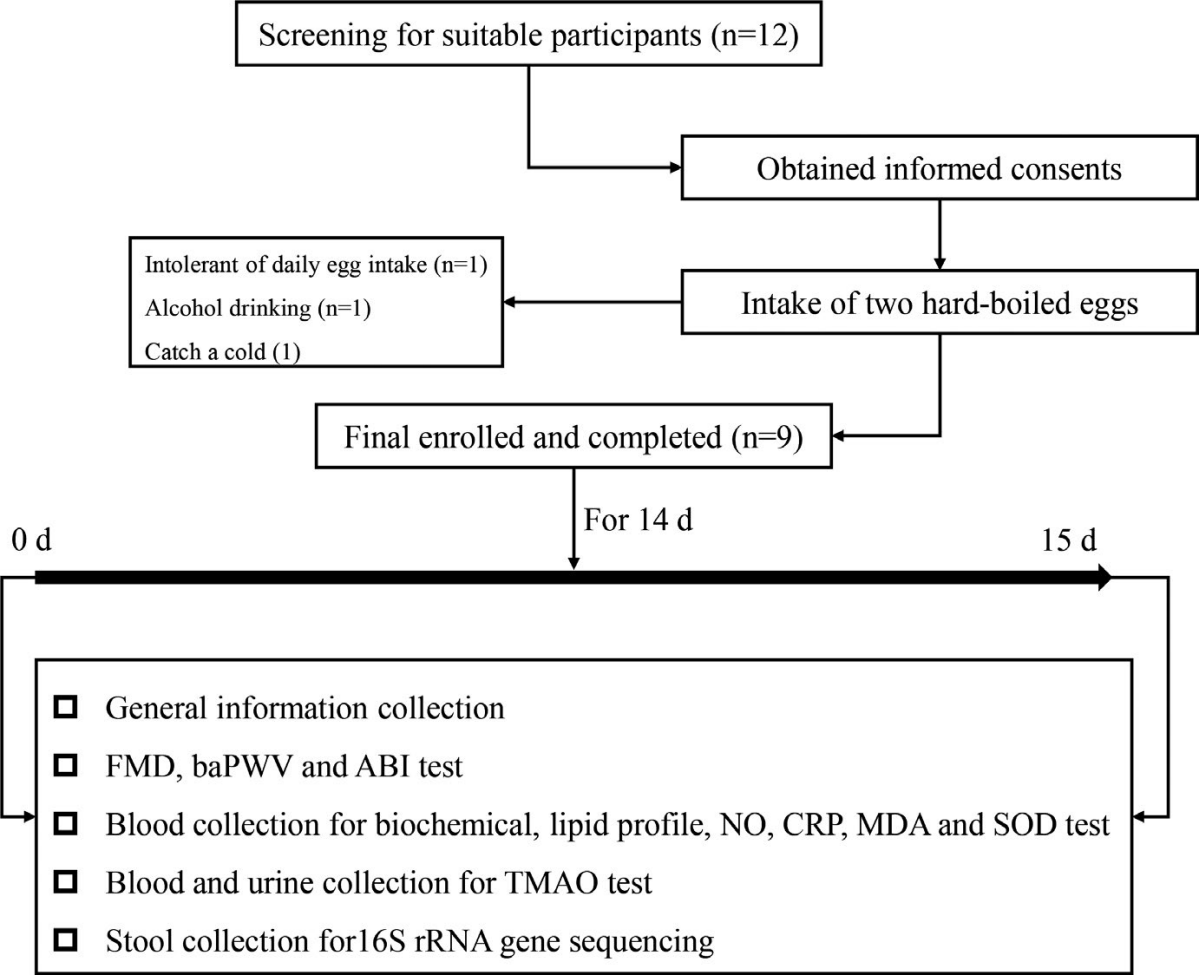
Flow chart of the study. Twelve subjects were enrolled, of which nine completed the trial. These subjects consumed two boiled eggs per day for 2 weeks, and changes in vascular function, inflammation, metabolism, oxidative stress, and gut microbiota were examined before and after the intervention

Twelve subjects were enrolled, of which nine completed the trial (Figure 1). The average age was 29 years, and the mean body mass index was 22 kg/m^2^. Participants consumed two boiled eggs on schedule, and no intervention‐related adverse events were reported. There were no intervention‐related differences in heart rate, blood pressure, CRP, glucose, creatinine, or lipid profile. The clinical characteristics and laboratory results are summarized in Table 1.

**TABLE 1:** Subject characteristics and laboratory indices at baseline and after intervention

Variables	Baseline	Two eggs	*p*
Age (yr)	29 ± 1	—	—
Male (*n*, %)	9 (100)	—	—
BMI (kg/m^2^)	22 ± 1	—	—
Heart rate (bpm)	62 ± 2	63 ± 2	0.643
SBP (mmHg)	116 ± 2	118 ± 3	0.118
DBP (mmHg)	69 ± 1	69 ± 2	0.908
Hs‐CRP (mg/L)	0.39 (0.24, 0.56)	0.30 (0.17, 0.91)	0.674
Fasting glucose (mmol/L)	4.3 ± 0.7	4.2 ± 0.1	0.137
Creatinine (μmol/L)	84 ± 6	82 ± 5	0.170
Uric acid (μmol/L)	396 ± 20	396 ± 26	0.991
ALT (U/L)	16 (13, 39)	15 (12, 28)	0.141
AST (U/L)	20 (18, 25)	22 (18, 24)	0.833
Triglycerides (mmol/L)	0.80 (0.65, 1.00)	0.90 (0.70, 1.05)	0.307
Total cholesterol (mmol/L)	4.61 ± 0.82	4.57 ± 0.68	0.685
HDL (mmol/L)	1.43 ± 0.25	1.36 ± 0.08	0.083
LDL (mmol/L)	2.87 ± 0.63	2.83 ± 0.56	0.740

Note

Values are presented as mean ± standard error of the mean or whole numbers and percentages or median and interquartile range.

Abbreviations: ALT, alanine aminotransferase; AST, aspartate aminotransferase; BMI, body mass index; DBP, diastolic blood pressure; HDL, high‐density lipoprotein; Hs‐CRP, high‐sensitivity C‐reactive protein; LDL, low‐density lipoprotein; SBP, systolic blood pressure.

### 3.2 Egg consumption improved vascular function

**FIGURE 2:**
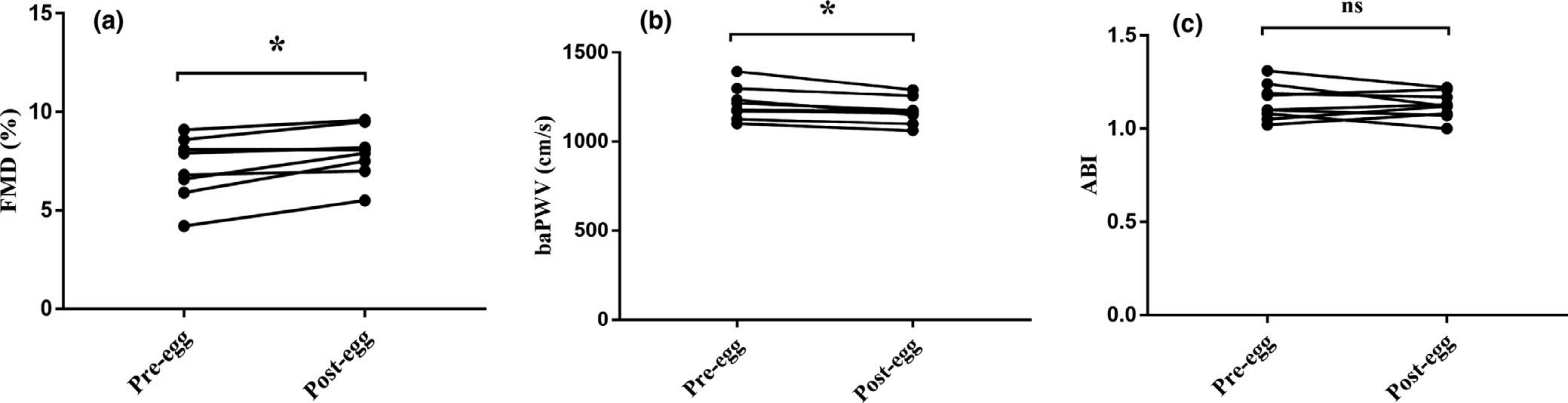
Egg consumption improved vascular function. (a) FMD was significantly enhanced, and (b) baPWV was decreased after the intervention, but (c) ABI remained unchanged. Data were analyzed by the paired sample *t* test. *n* = 9 per group. ^*^ *p* < .05 compared with pre‐egg. ABI, ankle‐brachial index; baPWV, brachial‐ankle pulse wave velocity; FMD, flow‐mediated dilation; ns, not significant; post‐egg, after egg intervention; pre‐egg, before egg intervention

FMD, baPWV, and ABI were measured before and after dietary intervention to investigate the effect of egg consumption on vascular function. Notably, egg consumption improved FMD (Figure 2a) and baPWV (Figure 2b) but did not affect ABI (Figure 2c).

### 3.3 Egg consumption did not influence oxidative stress markers and TMAO

**FIGURE 3:**
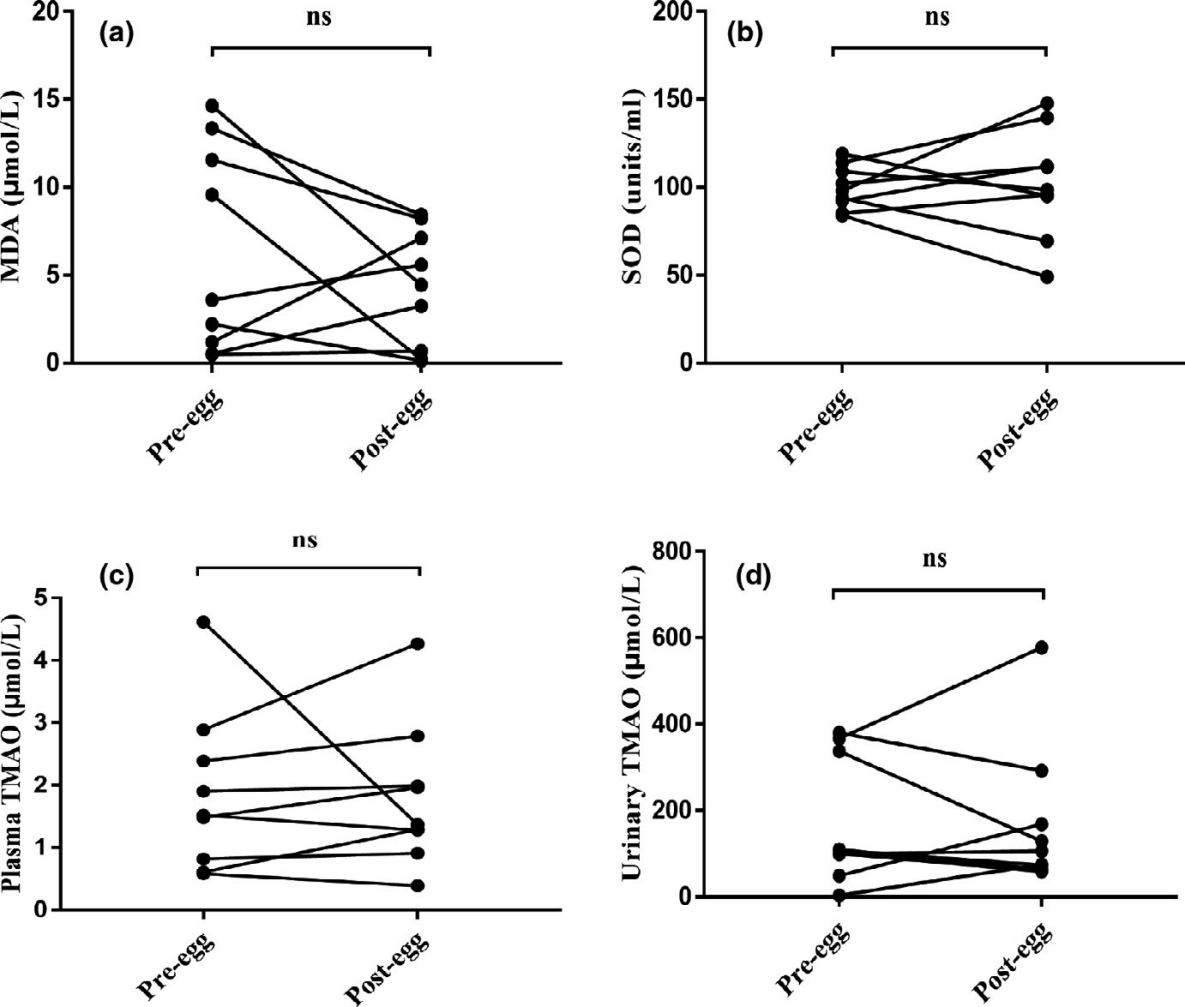
The effects of egg consumption on the levels of MDA, SOD, and TMAO. The plasma concentrations of (a) MDA, (b) SOD, (c) TMAO, and (d) urinary TMAO were not affected by egg consumption. Data were analyzed by the paired sample *t* test or Wilcoxon rank‐sum test. *n* = 9 per group. MDA, malondialdehyde; ns, not significant; post‐egg, after egg intervention; pre‐egg, before egg intervention; SOD, superoxide dismutase; TMAO, trimethylamine‐N‐oxide

Egg consumption did not affect MDA levels (pre: 6.35 ± 1.95 µmol/L, post: 4.22 ± 1.12 µmol/L) (Figure 3a) and SOD levels (pre: 99.73 ± 4.14 units/ml, post: 102.11 ± 10.32 units/ml) (Figure 3b). Neither plasma (pre: 1.52 [0.72, 2.64] μmol/L, post: 1.37 [1.10, 2.39] μmol/L) (Figure 3c) nor urinary TMAO (pre: 172.66 ± 48.71 µmol/L, post: 171.66 ± 56.39 µmol/L) was affected by egg consumption (Figure 3d).

### 3.4 Egg consumption did not alter the gut microbiota composition

**FIGURE 4:**
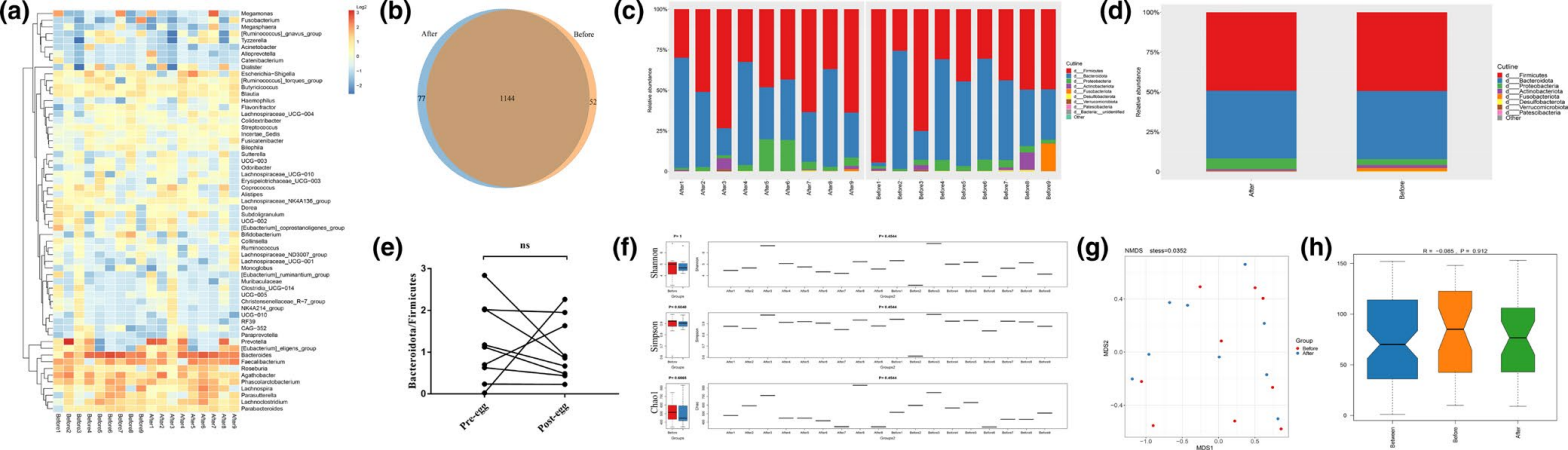
The effects of egg consumption on the gut microbiota composition. (a) The heatmap of different bacterial communities at the genus level is presented. (b) There are 1144 overlapping OTUs between the two groups, and 52 and 77 OTUs exclusively belong to the pre‐egg and post‐egg group, respectively. (c, d) The composition and abundance of the gut microbiota are presented at the phylum level, and (e) Firmicutes/Bacteroidota ratios remained unchanged. (f) The gut microbiota's alpha diversity and (g, h) beta diversity remained unchanged after egg consumption. Firmicutes/Bacteroidota ratios were analyzed by the paired sample *t* test. *n* = 9 per group. ns, not significant; OTU, operational taxonomic unit; pre‐egg, before egg intervention; post‐egg, after egg intervention

A heatmap of bacterial distribution illustrates the abundance of different communities at the genus level (Figure 4a). The Venn diagram shows 1144 overlapping OTUs and 52 and 77 OTUs exclusively belonging to the pre‐ and post‐intervention groups, respectively (Figure 4b). The taxonomic composition of the gut microbiota between the two groups was analyzed, and their abundances were presented at the phylum level (Figure 4c,d), in which Firmicutes/Bacteroidota ratios were not altered by egg intervention (Figure 4e). The alpha diversity of the gut microbiota was compared using the Shannon, Simpson, and Chao1 indexes, and it was found that the two groups had similar gut microbial alpha diversity (Figure 4f). Similarly, the Bray–Curtis distance matrix was used to generate an NMDS plot of beta diversity, and no differences were seen between groups (Figure 4g,h).

### 3.5 Egg consumption substantially altered the gut microbial function

**FIGURE 5:**
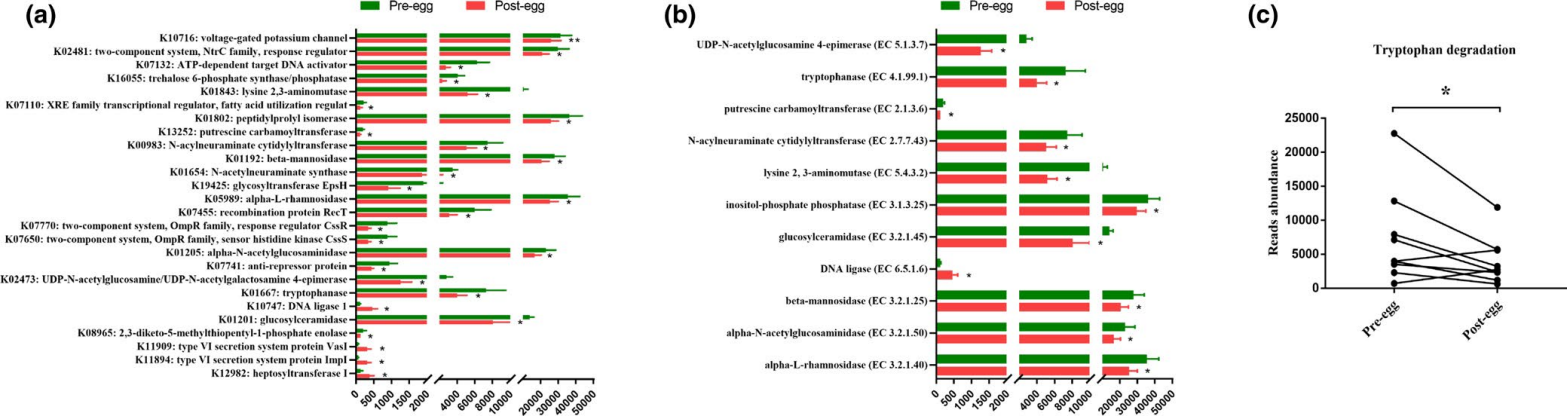
The effects of egg consumption on the function of gut microbiota. PICRUSt2 was adopted to conduct KO and EC prediction. (a) Egg intervention resulted in extensive down‐regulation of functional orthologs, except for DNA ligase 1, heptosyltransferase I, and type VI secretion system protein VasI and ImpI. (b) The gut microbiota of the post‐egg group was characterized by a remarkable reduction in enzyme EC abundance. (c) For functional modules (GMMs), the post‐egg group was less enriched in tryptophan degradation. Data were analyzed by the paired sample *t* test. *n* = 9 per group. ^*^ *p* < .05 compared with pre‐egg. EC, Enzyme Commission; GMMs, gut metabolic modules; KO, KEGG Ortholog; post‐egg, after egg intervention; pre‐egg, before egg intervention

PICRUSt2 was adopted to conduct KO (KEGG Ortholog) and EC (Enzyme Commission) prediction. Egg intervention resulted in downregulation of functional orthologs, including the voltage‐gated potassium channel pathway, ATP‐dependent target DNA activator pathway, and tryptophanase, etc. In contrast, DNA ligase 1, heptosyltransferase I, type VI secretion system protein VasI, and type VI secretion system protein ImpI were enriched in the post‐treatment group (Figure 5a). Moreover, the gut microbiota following egg intervention was characterized by a significant reduction in enzyme EC abundance, such as α‐L‐rhamnosidase and α‐*N*‐acetylglucosaminidase, but not DNA ligase (Figure 5b). For GMMs, the post‐egg group was less enriched in tryptophan degradation (Figure 5c).

## 4 DISCUSSION

In this study, we found that a daily intake of two eggs improved FMD and baPWV and positively modulated the function of the gut microbiota but exerted no effects on CRP, glucose, lipid profile, MDA, SOD, or TMAO. To our knowledge, this is the first study to evaluate the impact of hard‐boiled egg intake on vascular function, metabolism, inflammation, oxidative stress, and gut microbiota in healthy, young male subjects. The results suggest that moderate egg consumption may improve vascular and intestinal function in individuals with low risk of developing CVD and other metabolic disorders.

Eggs are rich in essential proteins, nutrients, vitamins, and minerals, and high in cholesterol and choline (Andersen, 2015). The benefits of egg consumption are well documented in the literature (Matsuoka et al., 2018; Ramli et al., 2020). However, there is still controversy about the effects of egg consumption on CVD risk. In a study conducted with 29,615 adult participants, researchers found that a higher dietary cholesterol or eggs intake was related to an increased risk of CVD and all‐cause mortality in a dose‐dependent manner (Zhong et al., 2019). On the contrary, in a cohort study of over 500,000 Chinese adults, researchers found that daily egg intake was associated with a decrease in CVD risk compared with those who had never or rarely consumed eggs (Qin et al., 2018). In another study among Chinese individuals, egg intake was associated with a lower total mortality, and it was found that other sources of cholesterol may negatively impact longevity (Zhuang et al., 2020).

Due to the inconsistencies in previous studies, the goal of this study was to determine the relationship between egg intake and CVD risk. First, we evaluated FMD, baPWV, and ABI at baseline and after egg intervention. We found that egg consumption significantly improved FMD and baPWV, indicating that egg consumption improves vascular function. It has been widely accepted that damage to the vascular endothelium leads to endothelial dysfunction, which is an early sign of atherogenesis (Gimbrone & Garcia‐Cardena, 2016). FMD has been extensively used to evaluate endothelium‐dependent vasodilation (Joannides et al., 1995; Thijssen et al., 2019). In addition, arterial stiffness, which can be assessed by baPWV, is an early indication of impaired vascular wall structure and function. Arterial stiffness is associated with an increased risk of developing heart failure, hypertension, coronary artery disease, diabetes mellitus (Boutouyrie et al., 2021; Ikonomidis et al., 2015; Prenner & Chirinos, 2015; Reddy et al., 2017), and a significant increase in CVD risk (Mitchell et al., 2010).

Egg yolks are high in cholesterol, and egg intake may contribute to elevated plasma lipid levels. According to a recent study, a high intake of egg (with a mean consumption of 136.77 g/day) was associated with increased total cholesterol, LDL‐cholesterol, and non‐HDL‐cholesterol, but exerted a beneficial effect on HDL‐C and triglycerides (C. Liu et al., 2020). These results were further supported by a similar conclusion drawn from a meta‐analysis (Rouhani et al., 2018). However, other studies have found no correlation between egg consumption and blood lipid levels, CVD, and mortality (Dehghan et al., 2020; M. X. Wang et al., 2019). Consistent with these findings, in our present work, we showed that triglycerides, total cholesterol, HDL, and LDL were not significantly affected by egg intake. We also found that egg consumption did not affect CRP, MDA, and SOD levels, suggesting that inflammation and oxidative stress were not involved. One study has found that regular egg consumption improves antioxidant status in young women (Taguchi et al., 2018). Furthermore, egg intervention did not affect TMAO levels in plasma and urine, which is consistent with previous studies (DiMarco et al., 2017; Missimer et al., 2018; Zhu et al., 2020).

Increasing evidence shows that the interactions between food and gut microbiota play an essential role in health and diseases (Danneskiold‐Samsoe et al., 2019), and a high‐fat diet can contribute to dysbacteriosis (Wei et al., 2020). Furthermore, studies revealed that egg consumption improves the homeostasis of intestinal flora (Meng et al., 2020; H. Yu et al., 2020). In a study of postmenopausal women, a daily intake of two eggs did not affect the gut microbiome composition, and considerable interindividual variability existed (Zhu et al., 2020). In the present study, we investigated the effects of egg consumption on gut microbiota. It was concluded that the function of gut microbiota was substantially altered, despite the absence of relevant changes in the taxonomic composition, alpha diversity, and beta diversity. Similarly, another study showed no changes in the gut microbiome composition of LDLR knockout mice following a high cholesterol diet (Dimova et al., 2017). Bacteria from the phyla Firmicutes and Bacteroidetes account for most of the gut microbiota (Stojanov et al., 2020), and increased Firmicutes/Bacteroidetes ratio plays a role in obesity (Stojanov et al., 2020; Wu, Gao, et al., 2020). In our current study, Firmicutes/Bacteroidetes ratio remained unchanged after egg intervention, suggesting that intestinal homeostasis was maintained. In contrast, egg consumption led to less tryptophan degradation enrichment. Enhanced degradation of tryptophan has been linked to inflammation, and maintaining the homeostasis of tryptophan degradation may be a therapeutic target in patients with CVD (G. Liu et al., 2017). In mitogen‐stimulated human peripheral blood mononuclear cells, cacao extracts suppress inflammation by reversing the degradation of tryptophan (Jenny et al., 2009). Similarly, an increase in plasma tryptophan levels was associated with reduced CVD risk (E. Yu et al., 2017). Moreover, KOs related to DNA ligase 1 and type VI secretion system were enriched in the post‐egg group. DNA ligase 1 plays an essential role in DNA replication and repair (Howes & Tomkinson, 2012; Liddiard et al., 2019), and the Type VI secretion system exists widely in gram‐negative bacteria and plays a role in bacterial competition and interaction (Coulthurst, 2019; Trunk et al., 2018). Collectively, these results suggest that egg consumption exerts a beneficial effect on intestinal function, which may be responsible for the improved vascular function; however, the exact mechanism remains unclear.

This study has several limitations. Firstly, with a sample size of nine, this study may not provide conclusive evidence. Secondly, the duration of the intervention is short, which may partially contribute to the lack of change observed in TMAO levels and gut microbiota composition. Thirdly, the exact mechanism by which egg consumption improved vascular function remains unclear. Finally, the relationships between vascular function and gut microbiota are not known. Therefore, further research is warranted to explore the underlying mechanism by which egg consumption improves vascular health and gut microbiota function and how these are related to each other.

## 5 CONCLUSION

The current study reveals that a daily intake of two eggs for 2 weeks significantly improved FMD and baPWV and positively affected intestinal function. There were no changes in metabolism, inflammation, and oxidative stress. In summary, this study shows that moderate egg consumption may help to improve vascular and intestinal function in individuals at low risk of developing CVD and other metabolic disorders.

## CONFLICTS OF INTEREST

The authors declare no conflict of interest.

## AUTHOR CONTRIBUTION

Xiang Liu: Conceptualization (equal); Formal analysis (equal); Investigation (equal); Writing—original draft (equal). Yijia Shao: Formal analysis (equal); Investigation (equal); Project administration (equal). Jiapan Sun: Formal analysis (equal); Investigation (equal). Jiazichao Tu: Investigation (equal). Zhichao Wang: Investigation (equal). Jun Tao: Conceptualization (equal); Funding acquisition (equal); Supervision (equal); Writing—review & editing (equal). Jimei Chen: Conceptualization (equal); Data curation (equal); Funding acquisition (equal); Supervision (equal); Writing—review & editing (equal).
